# Catalytic mTOR inhibitors can overcome intrinsic and acquired resistance to allosteric mTOR inhibitors

**DOI:** 10.18632/oncotarget.2337

**Published:** 2014-08-10

**Authors:** Burhan Hassan, Argun Akcakanat, Takafumi Sangai, Kurt W. Evans, Farrell Adkins, Agda Karina Eterovic, Hao Zhao, Ken Chen, Huiqin Chen, Kim-Anh Do, Shelly M. Xie, Ashley M. Holder, Aung Naing, Gordon B. Mills, Funda Meric-Bernstam

**Affiliations:** ^1^ Department of Surgical Oncology, The University of Texas MD Anderson Cancer Center, Houston, TX; ^2^ Department of Investigational Cancer Therapeutics, The University of Texas MD Anderson Cancer Center, Houston, TX; ^3^ Department of Systems Biology, The University of Texas MD Anderson Cancer Center, Houston, TX; ^4^ Department of Bioinformatics and Computational Biology, The University of Texas MD Anderson Cancer Center, Houston, TX; ^5^ Department of Biostatistics, The University of Texas MD Anderson Cancer Center, Houston, TX

**Keywords:** mTOR, Akt, rapamycin, everolimus, breast cancer

## Abstract

We tested the antitumor efficacy of mTOR catalytic site inhibitor MLN0128 in models with intrinsic or acquired rapamycin-resistance. Cell lines that were intrinsically rapamycin-resistant as well as those that were intrinsically rapamycinsensitive were sensitive to MLN0128 *in vitro*. MLN0128 inhibited both mTORC1 and mTORC2 signaling, with more robust inhibition of downstream 4E-BP1 phosphorylation and cap-dependent translation compared to rapamycin *in vitro*. Rapamycin-sensitive BT474 cell line acquired rapamycin resistance (BT474 RR) with prolonged rapamycin treatment *in vitro*. This cell line acquired an mTOR mutation (S2035F) in the FKBP12-rapamycin binding domain; mTORC1 signaling was not inhibited by rapalogs but was inhibited by MLN0128. In BT474 RR cells, MLN0128 had significantly higher growth inhibition compared to rapamycin *in vitro* and *in vivo*. Our results demonstrate that MLN0128 may be effective in tumors with intrinsic as well as acquired rapalog resistance. mTOR mutations are a mechanism of acquired resistance *in vitro*; the clinical relevance of this observation needs to be further evaluated.

## INTRODUCTION

The Phosphatidylinositol 3-kinase (PI3K)/Akt/ mammalian target of rapamycin (mTOR) pathway plays a central role in cell metabolism, growth, proliferation and, survival [1, 2]. Activation of PI3K/Akt/mTOR signaling contributes to the pathogenesis of many tumor types, and thus the pathway is being actively pursued as a promising therapeutic target [3-5]. mTOR, an important component of this pathway, exists in two multiprotein complexes: mTOR complex 1 and mTOR complex 2 (mTORC1 and mTORC2). mTORC1 includes the mTOR protein, mammalian LST8, proline rich Akt substrate 40 (PRAS40) and raptor [1, 6, 7] and controls cell growth, survival, angiogenesis and protein translation via its two major substrates S6 kinase (S6K) and 4E-BP1 [8, 9]. Activated S6K causes feedback inhibition of insulin-like growth factor-1 (IGF-1)/insulin signaling by phosphorylating insulin receptor substrate 1 (IRS-1) resulting in its degradation [10]. mTORC2 consists of mTOR, mLST8, mSIN1, protor and rictor [11-15]. It has been shown that mTORC2 phosphorylates Akt at Serine 473 (S473), enhancing the catalytic activity of Akt, that has already been phosphorylated at Threonine 308 (T308) [16, 17]. Thus, the mTOR complexes play an important role both upstream and downstream of Akt [18].

Rapamycin and its analogs are allosteric mTOR inhibitors that bind FKBP12 and mTOR, and predominantly inhibit mTORC1. The rapamycin analog temsirolimus is approved by the Food and Drug Administration for the treatment of advanced renal cell carcinoma and the rapamycin analog everolimus is FDA approved for the treatment of pancreatic neuroendocrine tumors, renal cell carcinoma, sub-ependymal giant cell astrocytoma associated with tuberous sclerosis, as single agent therapy, and for the treatment of hormone-receptor positive breast cancer as combination therapy with exemestane. Rapalogs have also shown promise in clinical trials in other tumor types, such as mesothelioma and endometrial cancer [1, 19]. However, rapalogs have shown objective responses in only a subset of patients, and unfortunately the responses are frequently short-lived. Mechanisms of acquired resistance to rapalogs are unknown.

These therapeutic failures have been attributed, in part, to rapamycin-induced Akt activation, as a result of inhibition of the S6K/IRS-1 feedback loop. Rapamycin not only inhibits S6K phosphorylation but also induces Akt S473 phosphorylation, hence activating Akt [20, 21]. Although we have observed that rapamycin-induced Akt phosphorylation is increased more in rapamycin-sensitive cell lines compared to resistant cell lines [3], rapamycin-mediated Akt activation may be responsible for the attenuated antitumor efficacy of rapamycin and its analogs observed in patients. Approaches to prevent Akt activation, such as the use of inhibitors of upstream signaling, are being pursued [22]. However, an alternate approach is to target this pathway with mTOR kinase inhibitors that potently inhibit mTORC1 as well as mTORC2, thus inhibiting Akt S473 phosphorylation, and thereby preventing or attenuating the feedback loop activation of Akt and potentially treating PI3K/mTOR dependent cancers more effectively [23].

MLN0128, also known as INK128, is a novel ATP-competitive mTOR kinase inhibitor, currently in phase I clinical trials for advanced solid malignancies. We sought to determine the effect of MLN0128 on rapamycin sensitive cell lines as well as in cell lines with intrinsic and acquired rapamycin-resistance both *in vitro* and *in vivo*. We demonstrate that MLN0128 caused greater inhibition of mTORC1 signaling, mTORC2 signaling, cell cycle progression and translation in most cell lines compared to rapamycin. Likewise, MLN0128 sensitivity was significantly greater in cell lines that have either intrinsic resistance to rapamycin or have acquired rapamycin resistance highlighting the efficacy of this compound in resistant settings compared to rapamycin. Furthermore, MLN0128 caused growth inhibitory effect in several *in vivo* models, and had a greater growth-inhibitory effect in *in vivo* models of acquired rapamycin resistance. Further, we report that prolonged rapamycin treatment *in vitro* was associated with acquisition of an mTOR kinase mutation, with insensitivity to rapamycin-mediated inhibition of mTOR signaling and cell growth, but with sensitivity to MLN0128.

## RESULTS

### MLN0128 has Potent Antitumor Efficacy *In Vitro*

**Figure 1:**
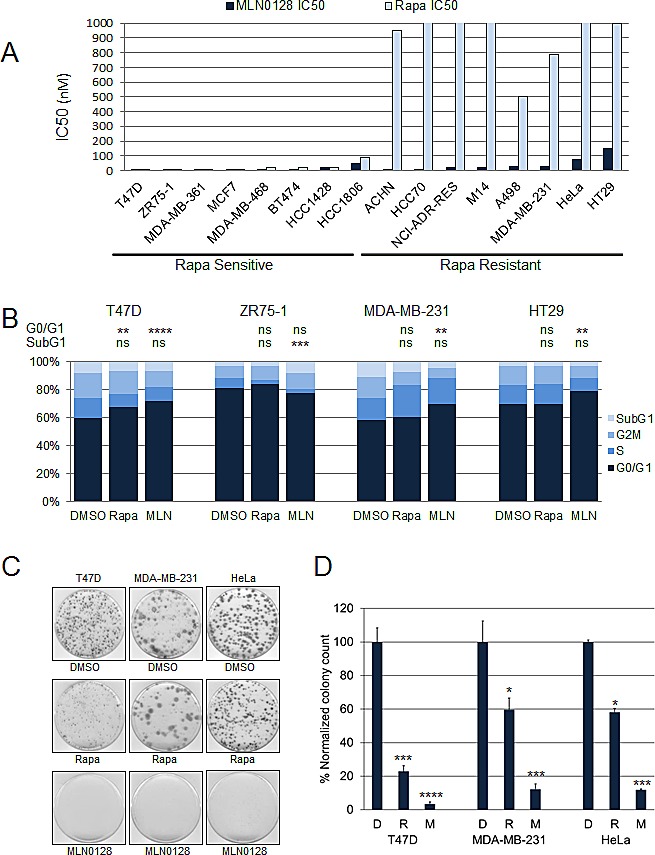
MLN0128 has potent antitumor efficacy *in vitro* (A) Sixteen cell lines with varying genetic backgrounds were treated with increasing doses of MLN0128 and rapamycin with IC50 being determined by SRB assay. (B) Cancer cell lines were treated with vehicle, rapamycin (100 nM), or MLN0128 (100 nM) in triplicate for 96 hours, and percentages of cells in G1 (navy), S (royal blue), G2/M (blue), and SubG1 (light blue) phases of the cell cycle were determined by flow cytometry. The percentages of cells in G1 or subG1 phases in each treatment group were compared (**P<0.01, ***P<0.001, ****P<0.0001, ns not significant, vs. control). This experiment was repeated three times and the results of one representative experiment done in triplicates are shown. (C) Effect of MLN0128 treatment on anchorage-dependent growth. T47D, MDA-MB-231, and HeLa cells were trypsinized, counted and plated at a density of 0.5-1 × 10^3^ cells/60 mm plates in triplicate for each treatment group. Cells were treated with vehicle, rapamycin (100 nM), or MLN0128 (100 nM) in triplicate for 2-3 weeks, colonies were then stained with crystal violet. (D) Individual colonies were counted using NIH ImageJ v.1.46 software. The colonies in each treatment group were normalized and compared. Data are presented as mean ± SE (*P<0.05, ***P<0.001, ****P<0.0001, vs. control). This experiment was repeated three times and the results of one representative experiment done in triplicates are shown.

We tested the MLN0128 sensitivity of 16 cell lines; the panel was enriched for breast cancer cell lines but consisted of cell lines with varying genomic alterations including mutations in *PIK3CA* and *PTEN*. These cell lines were selected as they represented cell lines with a range of sensitivities to rapamycin based on our previous study of a larger 43 cell line screen for rapamycin sensitivity (Supplementary Table S1) [3]. MLN0128 and rapamycin sensitivity was assessed by sulforhodamine B (SRB) assay (Fig. 1A). As expected the sensitivity of cell lines to rapamycin varied [3]. Most cell lines were sensitive to MLN0128 with IC50s in the low nano-molar range. T47D and ZR75-1 cell lines were sensitive to both MLN0128 and rapamycin. MDA-MB-231, ACHN, and A498 cell lines were resistant to rapamycin but sensitive to MLN0128. HT29 and HeLa cell lines were resistant to rapamycin, and were less sensitive to MLN0128, but still had significant growth inhibition with clinically achievable MLN0128 concentrations (HT29 IC50=150 nM and HeLa IC50=75 nM).

The effect of MLN0128 on cell cycle progression was analyzed by flow cytometry (Fig. 1B). Cancer cell lines were treated with vehicle, rapamycin 100 nM, or MLN0128 100 nM for 3 days, and percentages of cells in subG1, G1, S, and G2/M phases were measured. Both MLN0128 (*P*<0.0001) and rapamycin (*P*<0.01) caused significant inhibition of cell cycle progression from G1 to S phase in T47D. In MDA-MB-231 and HT29, MLN0128, but not rapamycin, significantly increased the percentage of cells in G1 phase (*P*<0.001). In ZR75-1 cell lines neither treatment increased cells in G1 phase; however, MLN0128 caused a significant increase in the subG1 population of cells (*P*<0.001).

To determine whether MLN0128 induced apoptosis, cancer cell lines representative of each group were treated with vehicle or rapamycin 100 nM or MLN0128 100 nM for 3 days, and the percentages of annexin V positive cells were determined (Supplementary Fig. S1A). MLN0128 induced significant apoptosis (*P*<0.0001) in ZR75-1 cells only, corresponding with an increase in the subG1 population of cells observed in cell cycle analysis. We did not see significant apoptosis in other cell lines. Immunoblotting also showed no increase in expression of cleaved caspase 3 or cleaved PARP with MLN0128 treatment (Supplementary Fig. S1B).

We also assessed the effect of MLN0128 and rapamycin on anchorage-dependent growth of T47D, MDA-MB-231, HeLa and HT29 cells using a colony formation assay. Two weeks later, cell colonies were stained with crystal violet, plates were scanned and the colonies quantitated. MLN0128 treatment resulted in a dramatic decline in colony-forming ability compared with rapamycin treatment (Fig. 1C and D).

Hence, MLN0128 was found to have growth inhibitory in several cancer cell lines with differing genomic backgrounds and with varying rapamycin sensitivity. However, MLN0128 predominantly had a cytostatic effect with little effect on apoptosis and or autophagy in most cell lines tested.

### MLN0128 Inhibits mTORC1 and mTORC2 Signaling

**Figure 2:**
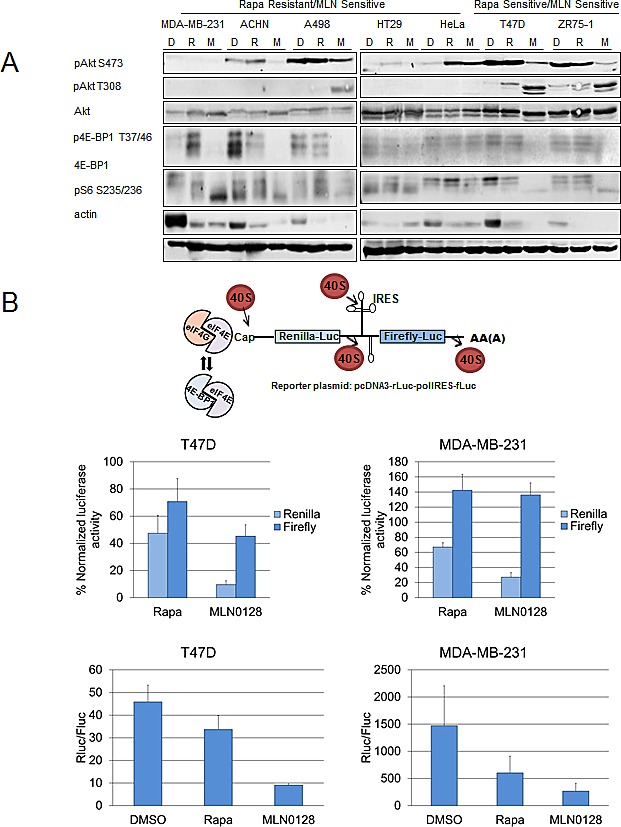
MLN0128 inhibits mTORC1 and mTORC2 signaling (A) MDA-MB-231, ACHN, A498, HT29, HeLa, T47D, and ZR75-1 cell lines were treated daily with vehicle, rapamycin (100 nM), and MLN0128 (100 nM) for 72 hours. Western blotting was performed to assess mTORC1 and mTORC2 signaling. (B) MLN0128 decreases translational activity. Cancer cell lines T47D and MDA-MB-231 were trypsinized, counted, and plated on 60 mm plates. They were transfected with a bicistronic pcDNA3-rLuc-polIRES-fLUC plasmid (construct detailed in the figure, where Renilla-Luc measures cap-dependent activity and Firefly-Luc measures cap-independent activity) and then treated with vehicle, rapamycin (100 nM), and MLN0128 (100 nM) for 24 hours. Dual luciferase assay kit was used to measure luciferase activity. The data represent the mean ± SE of three independent experiments performed in triplicate and analyzed (**P<0.01, ***P<0.001, vs. control).

mTOR kinase inhibitors have been shown to inhibit mTORC1 and mTORC2 [24, 25]. The effects of MLN0128 and rapamycin on mTORC1 and mTORC2 signaling were compared in rapamycin sensitive and resistant cell lines groups (Fig. 2A). Rapamycin potently inhibited the phosphorylation of S6, downstream substrate of mTORC1, but poorly inhibited 4E-BP1 phosphorylation as has been previously described [26, 27]. In contrast MLN0128 effectively inhibited S6 and 4E-BP1 phosphorylation, hence showing strong mTORC1 inhibition compared with rapamycin.

As has been reported previously, rapamycin did not inhibit mTORC2 and in certain contexts, induced Akt S473 phosphorylation [20, 21]. On the contrary, MLN0128 inhibits phosphorylation of Akt at S473, a downstream substrate of mTORC2 (Fig. 2A). Hence MLN0128 inhibited mTORC2, preventing mTORC2-dependent feedback activation of Akt; however, MLN0128 did increase phosphorylation of Akt at T308 in T47D, ZR75-1, and A498 cells (Fig. 2A).

### MLN0128 Decreases Cap-dependent and Cap-independent Translational Activity

To determine the effect of MLN0128 on protein translation, a reporter plasmid construct pCDNA3-rLuc-polIRES-fLUC was used that once transfected measures cap-dependent translational activity through renilla luciferase activity and internal ribosomal entry system (IRES) dependent translation through firefly luciferase activity. T47D (intrinsically sensitive to rapamycin) and MDA-MB-231 cells (intrinsically resistant to rapamycin), were transfected with reporter plasmid and a dual luciferase assay kit was used to measure renilla and firefly activity (Fig. 2B). In both cell lines, MLN0128 treatment decreased cap-dependent translation.

### MLN0128 Causes Growth Inhibition *In Vivo*

**Figure 3:**
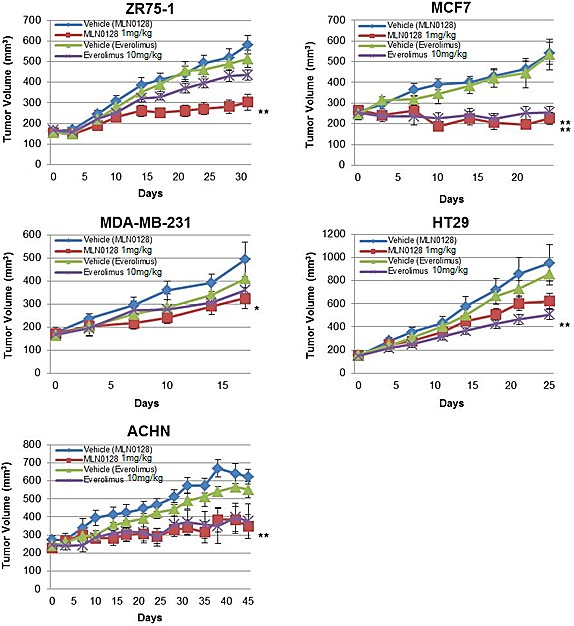
MLN0128 has *in vivo* antitumor efficacy Mice bearing ZR75-1, MCF7, MDA-MB-231, HT29, and ACHN xenografts were treated with vehicle, everolimus 10 mg/kg, and MLN0128 1 mg/kg. The tumor volumes at the conclusion of experiment were compared to vehicle and data was analyzed by Mann-Whitney U test. Data are presented as mean ± SE (*P<0.05, **P<0.01, vs. control).

To expand our findings *in vivo*, we determined the activity of MLN0128 in five different xenograft models. We analyzed xenografts from ZR75-1, MCF7 cell lines that are rapamycin-sensitive *in vitro* and xenografts from ACHN, MDA-MB-231 and HT29 cell lines that are rapamycin-resistant *in vitro*. The statistical analyses were done by comparing tumor volumes in treatment arms with tumor volumes in the vehicle arm at the termination of experiment. Mice bearing ACHN, ZR75-1 or MDA-MB-231 xenografts did not show statistically significant growth inhibition with everolimus treatment (*P*=0.1792, *P*=0.0591, and *P*=0.8541 respectively) (Fig. 3). In contrast, MLN0128 treatment, at doses of 1 mg/kg, previously described as an *in vivo* effective dose [28-31], led to significant tumor growth inhibition was observed compared with vehicle in all three cell lines (ACHN *P*=0.0093, ZR75-1 *P*=0.0012, MDA-MB-231 *P*=0.0263). In MCF7 xenografts, both MLN0128 and everolimus caused significant growth inhibition (*P*=0.0012 and *P*=0.0022, respectively). In contrast, HT29 xenografts responded to everolimus resulting in significantly lower tumor volume (*P*=0.0072) but with MLN0128 treatment, a decrease in tumor volume was observed but was not statistically significant (*P*=0.0827).

### MLN0128 is Effective in Cell Lines with Acquired Resistance to Rapamycin

Although rapalogs often have antitumor efficacy of limited duration in the clinic, currently little is known about mechanisms of acquired resistance to rapalogs. To get insight into mechanisms of acquired rapamycin resistance and approaches to overcome them, we created BT474 rapamycin resistant (BT474 RR) cell lines through culturing rapamycin-sensitive BT474 parental cells (BT474 Par) in progressively higher concentrations of rapamycin. We then tested the activity of MLN0128 in BT474 Par and RR cell lines *in vitro*.

**Figure 4:**
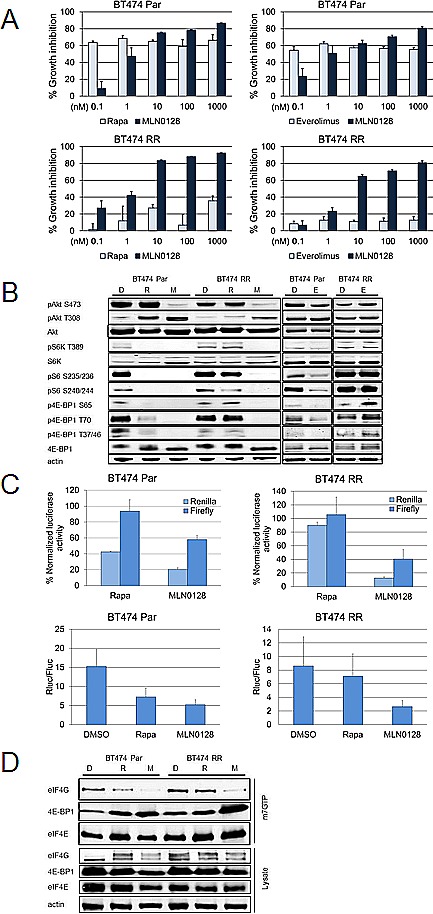
MLN0128 is effective in cell lines with acquired rapamycin resistance (A) BT474 Par and RR cell lines were treated with increasing doses of MLN0128, everolimus and rapamycin, using SRB assay to determine IC50. These experiments were repeated at least three times and data are presented as mean ± SE. (B) These cell lines were treated daily with vehicle, rapamycin (100 nM), and MLN0128 (100 nM) for 72 hours. Western blotting was performed to assess mTORC1 and mTORC2 signaling. (C) Cancer cell lines BT474 Par, and BT474 RR were trypsinized, counted and plated on 60 mm plates. They were transfected with pcDNA3-rLuc-polIRES-fLUC plasmid then treated with vehicle, rapamycin (100 nM), and MLN0128 (100 nM) for 24 hours. Dual luciferase assay kit was used to measure luciferase activity. The data represent mean ± SE of three independent experiments performed in triplicate and analyzed (**P<0.01, ***P<0.001, vs. control). (D) BT474 Par and RR cell lines were treated daily with vehicle, rapamycin (100 nM), or MLN0128 (100 nM) for 72 hours. Relative levels of cap-bound 4E-BP1 and eIF4G were compared using western blotting.

BT474 Par cell lines were sensitive to the growth-inhibitory effect of rapamycin and everolimus, and at clinically relevant levels, MLN0128 as well as, rapalogs, inhibited BT474 Par. In contrast neither rapamycin nor everolimus significantly inhibited BT474 RR cell line growth while MLN0128 demonstrated significant growth inhibitory effect on BT474 RR cells *in vitro* (Fig. 4A).

As expected in BT474 Par cells, immunoblotting showed that rapamycin inhibited mTORC1 substrates (p4E-BP1, pS6K) and downstream pS6, with activation of pAkt S473, while MLN0128 treatment inhibited pAkt S473 and inhibited p4E-BP1 more robustly (Fig. 4B). Strikingly in BT474 RR cell lines neither rapamycin nor everolimus inhibited the mTORC1 axis i.e. pS6, pS6K T389, or p4E-BP1. In contrast MLN0128 robustly inhibited mTORC1 signaling (Fig. 4A).

The effect of rapamycin and MLN0128 was then assessed on cap-dependent and independent translation. BT474 Par and BT474 RR cells were transfected with the bicistronic luciferase vector (as in Fig. 2B) and treated with rapamycin or MLN0128. In BT474 Par and BT474 RR cell lines, only MLN0128 caused statistically significant decline in both cap-dependent (both cell lines, *P*<0.001) and cap-independent (BT474 Par, *P*<0.01; BT474 RR, *P*<0.001) translational activity while rapamycin decreased cap-dependent (*P*<0.001) translational activity in BT474 Par cell lines only (Fig. 4C).

Phosphorylation of 4E-BP1 by mTORC1 leads to its dissociation from eIF4E, allowing binding of eIF4G at the same site, hence allowing translation initiation complex formation at the 5` end of mRNAs [27]. Thus, we expected that MLN0128 by de-phosphorylating 4E-BP1 leads to a decrease in translational activity, decreasing eIF4G-eIF4E binding. BT474 Par and BT474 RR cell lines were treated with control, rapamycin and MLN0128. The fraction of eIF4E associated with 4E-BP1 and eIF4G was examined by purification of lysates using 7-methyl GTP-sepharose beads; eIF4E and eIF4E-bound proteins were detected by immunoblotting analysis. Rapamycin led to a decrease in eIF4E-associated eIF4G in BT474 Par cells but not BT474 RR cells (Fig. 4D). In contrast, MLN0128 resulted in a decrease in eIF4G associated with eIF4E in both BT474 Par cells and BT474 RR cells.

### Rapamycin Resistant BT474 RR cells Harbor an mTOR Mutation

**Figure 5:**
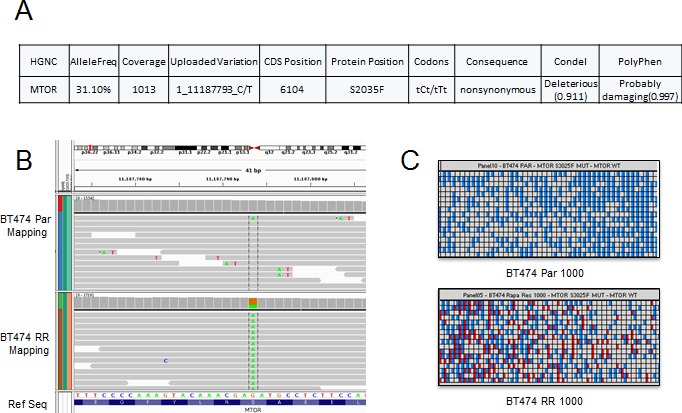
BT474 RR harbors an acquired mTOR mutation (A) Next-generation sequencing identified a mutation at mTOR S2035F in the resistant cell lines. (B) and (C) Heatmap view generated by the Biomark digital PCR analysis software for confirmation of mutation at mTOR S2035F. The panel shown here represents two samples: BT474 Par and BT474 RR 1000 copy/panel samples. The red spot highlights mutation.

As rapamycin did not inhibit mTORC1 signaling in BT474 RR cells, we hypothesized that prolonged rapamycin treatment may have caused an acquired mutation in mTOR or other upstream kinases with constitutive activation of mTORC1 signaling. To test this hypothesis, we performed targeted exome sequencing cells using a 202 gene panel that includes mTOR as well as multiple other cancer-related genes (Supplementary Table S2). Comparing BT474 Par and BT474 RR cells, we identified an mTOR S2035F mutation in the BT474 RR cells (Fig. 5A). This mutation corresponds to the FKBP12-rapamycin binding domain of mTOR and has been previously described *in vitro* transcription and translation assays, and in yeast models as a mutation known to interfere with mTOR-FKBP12 interaction and to confer rapamycin-resistance [32-34]. The existence of this point mutation was confirmed with digital polymerase chain reaction (Fig. 5B and 5C).

### Acquired Rapamycin-Resistant Cell Lines are Sensitive to MLN0128 *in vivo*

**Figure 6:**
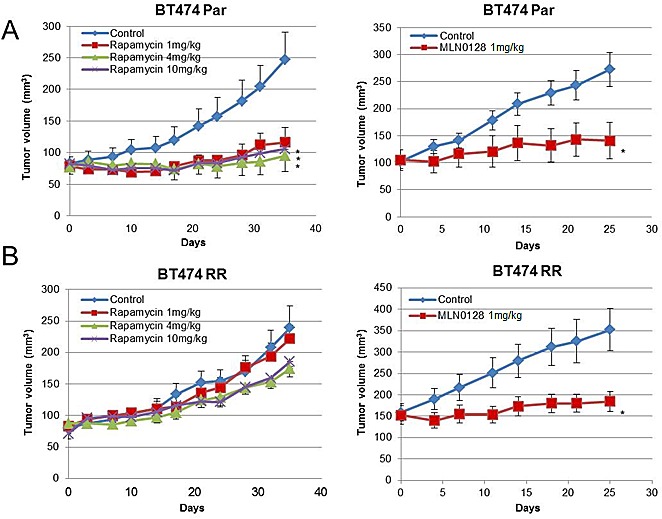
MLN0128 inhibits *in vivo* growth of rapamycin resistant BT474 RR cells (A) Mice bearing BT474 Par xenografts were treated with vehicle, rapamycin 1 mg/kg, 4 mg/kg, 10 mg/kg, or MLN0128 1 mg/kg. (B) Mice bearing BT474 RR xenografts were treated with vehicle, rapamycin 1 mg/kg, 4 mg/kg, 10 mg/kg, or MLN0128 1 mg/kg. Tumor volumes at the conclusion of the experiment were compared to vehicle, and data was analyzed by Mann-Whitney U test Data are presented as mean ± SE (*P<0.05).

Next, we determined the *in vivo* effect of rapamycin and MLN0128 in BT474 PAR and RR xenograft models. In the BT474 Par xenografts, both rapamycin and MLN0128 treatment showed significant tumor growth inhibition (for all treatment groups, *P*<0.05) compared to vehicle (Fig. 6A and 6B). In the BT474 RR xenograft model, rapamycin treatment did not lead to significant tumor growth inhibition; however, MLN0128 treatment resulted in significant tumor growth inhibition (*P*=0.0152) in comparison with vehicle.

## DISCUSSION

Akt/mTOR signaling plays key roles in controlling major cellular processes, including cell growth, protein translation, autophagy, metabolism, and cell survival [1, 2]. Activated Akt/mTOR signaling is a significant contributor to pathogenesis of cancer. Akt and mTOR have been shown to reciprocally regulate activity [25]. MLN0128 is an ATP-competitive mTOR kinase inhibitor; we sought to determine the antitumor efficacy of MLN0128 in cell lines of varying genetic backgrounds and varying sensitivity to rapamycin. We demonstrated that MLN0128 potently inhibits both S6 and 4E-BP1 phosphorylation in cells, with more robust inhibition of mTORC1 signaling than rapamycin; also in addition, MLN0128 completely inhibits the phosphorylation of Akt S473, consistent with its efficient inhibition of mTORC2 as well.

Rapamycin analogs have been FDA-approved for treatment of several tumor types, but single agent treatment has resulted in modest objective response rates. Where there is activity observed with allosteric mTOR inhibitors, they appear to be cytostatic, primarily stabilizing clinical disease, rather than resulting in tumor regression [1]. mTORC1 is implicated in several human diseases, such as diabetes, heart disease, obesity, and cancer. These diseases reveal aberrant cell growth and proliferation. Unlike S6Ks, 4E-BPs do not have an effect on cell size, but they regulate proteins involved in cell proliferation and cell cycle progression [35]. By evolving resistance to mTOR inhibitors, several studies identified a decrease in 4E-BP expression and an increase in expression of eIF4E and c-Myc [36-38]. Further, the 4E-BP/eIF4E ratio was suggested as an indicator of acquired and intrinsic resistance [36]. In BT474 Par cells, we observed a partial inhibition of phosphorylation of 4E-BP1 by rapamycin and everolimus, whereas in BT474 RR cells there was no inhibition. In contrast, MLN0128 inhibited 4E-BP1 phosphorylation in both cell lines completely. Considering the recently acknowledged importance of mTOR/4E-BP axis in rapamycin resistance, MLN0128 is likely to overcome this problem.

It has been proposed that this may be due to upregulation of feedback loops in which Akt phosphorylation and activity are increased by relieving S6K–driven suppression of IGF-1R signaling [21]. Some studies suggest that inhibition of mTORC2 will lead to the dephosphorylation of Akt at the S473 site and a more profound inhibition of Akt function than would be expected from dephosphorylation of Akt T308 alone [24, 39]. Thus, mTOR kinase inhibition has been proposed to prevent the feedback loop activation of Akt that may attenuate the response of patients with rapamycin therapy. In our data, MLN0128 showed potent and persistent mTORC1 and mTORC2 inhibition; however the inhibition of phosphorylation of Akt T308 is only temporary. These results are consistent with other work that demonstrated a biphasic Akt inhibition with mTOR kinase inhibitor AZD8055 [40]. This induction of PI3K activation may be due to relief of feedback activation of RTK signaling. Despite reactivation of Akt T308, MLN0128 was effective in inhibiting cell growth, and protein translation in cell lines that are not only sensitive to rapamycin but also in cell lines that are resistant to rapamycin. Most cell lines were sensitive to MLN0128 with an IC50 in nanomolar range *in vitro*. *In vivo*, MLN0128 resulted in significant growth inhibition in five of the xenograft models tested representing cell lines with a variety of genomic backgrounds (ZR75-1, MCF7, MDA-MB-231, BT474, ACHN, Supplementary Table S1). However, tumor regression was observed only in MCF7 xenografts emphasizing the need to pursue novel combinations, and raising the possibility that combinatorial therapies that also abrogate Akt T308 phosphorylation may have even greater *in vivo* tumor efficacy. We observed that some of our *in vitro* and *in vivo* results did not correlate completely. The growth of rapamycin-sensitive ZR75-1 xenografts was not significantly inhibited by everolimus. Similar discrepancies between *in vitro* and *in vivo* models are well-known. An in depth analysis of effect of everolimus on mTORC1 and mTORC2 activities may explain the mechanistic background of this discrepancy. Also, although HT29 cells were relatively resistant to rapamycin *in vitro*, the *in vivo* growth of HT29 xenografts was inhibited by everolimus (*P*=0.0072) but not by MLN0128 (*P*=0.0827). It is feasible that a larger study may have indeed demonstrated a statistically significant difference with MLN0128 as well. The greater antitumor efficacy *in vivo* than *in vitro* suggests that xenograft models capture additional antitumor mechanisms of action. Indeed a recently study but Mercier et al. demonstrated mTOR inhibition with rapamycin can have multiple effects on the microenvironment including decreases in the levels of angiogenesis, collagen deposition, and the total number of fibroblasts in the tumor stroma [41].

Most patients who derive clinical benefit from rapalogs may have disease stabilization for several months but ultimately progress. [41-43]The mechanism of acquired resistance to rapamycin analogs remains unknown at this time. There is increasing recognition that tumors evolve with progression and with treatment pressure. A variety of acquired genomic alterations that confer resistance to targeted therapies have been described including acquired Bcr-Abl mutations in response to imatinib treatment [44], ras mutations with cetuximab [45], EGFR mutations [46, 47] MET amplification with EGFR inhibitors[48], and loss of HER2 with trastuzumab-based therapy [49]. Thus, an acquired mutation in mTOR is a logical mechanism for acquired rapamycin resistance. This precise mutation has not been reported in TCGA or COSMIC [50]. However, an extensive study of serine at 2035 showed that substituting with aspartate, threonine, glutamine, and isoleucine abolished FKBP12-rapamycin binding, whereas conversion to alanine had a similar binding affinity compared to wild-type [33]. Also, these mTOR S2035 mutants can phosphorylate S6K and 4E-BP1 normally [34, 51, 52]. As there are no supporting experiments yet, it is not defined if the larger side chain of phenylalanine may abolish formation of complex or not. Further studies are needed to determine if this alteration is acquired in patients treated with rapalogs. If so, for patients who initially had benefited from rapalogs may achieve additional benefit from transitioning to an mTOR kinase inhibitor or another PI3K/Akt/mTOR inhibitor.

In summary, MLN0128 is an effective inhibitor of mTORC1 and mTORC2 activity. Cancer cell lines with intrinsic as well as acquired resistance to rapamycin are responsive to mTOR kinase inhibitor MLN0128. Ongoing clinical trials will test the efficacy of MLN0128 in patients with intrinsic as well as acquired resistance. We also report the novel finding of an acquired mTOR mutation associated with acquired rapamycin resistance. Further studies are needed to clinically validate this finding.

## METHODS

### Cell Lines and Cultures

Cancer cell lines obtained from American Tissue Culture Collection were: A498, ACHN (kidney), BT474, HCC70, HCC1428, HCC1806, MCF7, MDA-MB-231, MDA-MB-361, MDA-MB-435, MDA-MB-453, MDA-MB-468, T47D, ZR75-1 (breast), HeLa (cervix), HT29 (colon), and M14 (melanoma). NCI/ADR-RES ovarian cancer cells were obtained from the National Cancer Institute. All cell lines were maintained in DMEM/F12 media supplemented with 10% FBS and were passaged for less than 6 months after resuscitation and were authenticated by vendors.

### Acquired Resistance Cell Lines

The breast cancer cell line BT474 was cultured in progressively increasing doses of rapamycin until sustained growth at supra-therapeutic (10 μM) doses was achieved (approximately 18 months). Pool populations (non-clonal) of the parental cell lines (BT474 Par) and the cell lines with acquired rapamycin resistance (BT474 rapamycin resistant (RR)) were generated. Their identities were confirmed by short tandem repeat (STR) profiling.

### Reagents

Rapamycin was purchased from LC Laboratories., Inc. DMSO was bought from Sigma-Aldrich. MLN0128 for *in vitro* studies was kindly provided by Intellikine Inc. (La Jolla, CA). Additional MLN0128 was purchased from ChemieTek (Indianapolis, IN). For *in vivo* experiments methyl cellulose, polyvinylpyrrolidone and N-methyl-2-pyrrolidone (Sigma) were used as vehicles.

### Cell Growth Assay

Antiproliferative activity was tested by SRB assay [53]. The half maximal inhibitory concentration (IC50) was determined from dose-response curves after 4 days of treatment [54].

### Colony Formation Assay

Colony formation assay was performed as previously described [55]. Briefly, cells were trypsinized, counted and plated at a density of 1-5 × 10^3^ cells depending on the cell lines/60 mm plates in triplicate for each treatment group. Approximately 2-3 weeks later when the controls had colonies approaching confluency, plates were fixed, stained with crystal violet and scanned. The colonies were counted using NIH ImageJ v.1.46 software.

### Cell Cycle Analysis and Annexin V Binding Assay

For cell cycle assay, floating cells, as well those that were attached to the culture dish, were collected. Samples were analyzed by flow cytometry and ModFit LT software (Verity Software House). Apoptosis was identified by using the Annexin V apoptosis kit (Roche) according to the manufacturer's protocol, and cells were analyzed by flow cytometry and FlowJo v.10 (Tree Star) [54].

### Western Blotting

Immunoblotting was performed as described previously [56] with the following antibodies: Akt, pAkt T308, pAkt S473, caspase 3, eIF4E, eIF4G, LC3B, PARP, S6K, pS6K T389, pS6 S235/236, pS6 S240/244, 4E-BP1, p4E-BP1 T37/46, p4E-BP1 S65, p4E-BP1 T70 (Cell Signaling Technology, Inc.) and actin (Sigma).

### Dual Luciferase Assay

The reporter plasmid pCDNA3-rLuc-polIRES-fLUC was a gift from Nahum Sonenberg. T47D, MDA-MB-231, BT474 Par and BT474 RR cell lines were transfected with reporter plasmid using DharmaFECT transfection reagent by Thermo Fisher Scientific, Inc. (Waltham, MA). Dual luciferase assays were performed with the dual luciferase reporter assay system (Promega Corp., Madison, WI) according to the manufacturer's protocol [57].

### eIF4E-containing 5′ mRNA Cap Complex Analysis

BT474 Par and RR cells were treated with DMSO, rapamycin (100 nM) and MLN1028 (100 nM) for 72 hours. Approximately 500 μg of total protein for each condition was incubated with 7-methyl GTP Sepharose 4B beads (GE Healthcare, Piscataway, NJ) for two hours at 4°C. Pelleted beads were washed twice with lysis buffer and suspended in 1X SDS-PAGE sample buffer (containing 12.5% v/v β-mercaptoethanol) and eIF4E, eIF4G and 4E-BP1 levels contained in the elutes were analyzed by western blotting [58].

### Targeted Exome Sequencing

Genomic DNA was extracted from BT474 Par and BT474 RR cell lines using Qiagen's Qiaprep Kit according to the manufacturer's protocol. DNA was quantified by Qubit (Invitrogen) and quality was assessed using Genomic DNA Tape for the 2200 TapeStation (Agilent). DNA was sheared by sonication and to ensure the proper fragment size, samples were checked on TapeStation using the DNA High Sensitivity kit (Agilent). The sheared DNA proceeded to library prep using KAPA library prep kit (KAPA) following the “with beads” manufacturer protocol. Samples were quantified using KAPA qPCR quantification kit. Equimolar amounts of DNA were pooled (8-12 samples per pool) for capture of 202 genes that are clinically relevant in cancer (Supplementary Table S2). We designed biotin labeled probes with Roche Nimblegen for capturing target regions (all exons in those 202 genes) and followed manufacture's protocol for the capture step. The captured libraries were sequenced on a HiSeq 2000 (Illumina Inc., San Diego, CA, USA) on a version 3 TruSeq paired end flowcell according to manufacturer's instructions at a cluster density between 700 – 1000 K clusters/mm2. Sequencing was performed on a HiSeq 2000 for 2 × 100 paired end reads with a 7 nt read for indexes using Cycle Sequencing v3 reagents (Illumina). The resulting BCL files containing the sequence data were converted into “.fastq.gz” files and individual libraries within the samples were demultiplexed using CASAVA 1.8.2 with no mismatches. All regions were covered by >20 reads. For data analysis, the target-captured deep-sequencing data was aligned to human reference assembly hg19 using BWA and duplicated reads were removed using samtools. Single nucleotide variants (SNVs) and small indels were called using VarScan2 [59], which classified variants. To understand the potential functional consequence of detected variants, they were compared with dbSNP, COSMIC [50], and TCGA databases, and were annotated them using VEP [60], Annovar [61], SIFT [62], Polyphen [63], and Condel [64].

### Digital PCR

Genomic DNA was extracted and quantified as above. Custom multiplexed genotyping qPCR assays capable of distinguishing the wild type and mutant alleles of mTOR were designed by and ordered from Life Technologies. Following the manufacturer's (Fluidigm) protocol, 3.5 ng DNA was loaded on to a 12.75 Digital Array IFC chip and processed on the Fluidigm BioMark HD instrument. Data analysis was performed using Fluidigm's Digital PCR Analysis software.

### *In Vivo* Studies

All animal experiments were approved by the MD Anderson Animal Care and Use Committee. BT474 Par and BT474 RR (5×10^6^), MCF7 (5×10^6^), MDA-MB-231 (2×10^6^), and ZR75-1 (1×10^7^) cells were injected in the mammary fat pads of female nu/nu mice (Department of Experimental Oncology, MD Anderson), whereas HT29 (2×10^6^) and ACHN (1×10^7^) cells were injected subcutaneously. BT474 cell suspensions were mixed with Matrigel (BD Biosciences). Mice bearing ZR75-1, MCF7, BT474 Par, and BT474 RR xenografts were implanted with 17β-estradiol pellets (Innovative Research of America) subcutaneously. In a single agent treatment with rapamycin BT474 Par and BT474 RR xenografts mice were randomized into 4 groups (vehicle, rapamycin 1 mg/kg, rapamycin 4 mg/kg, or rapamycin 10 mg/kg, once weekly by intraperitoneal injection, n=8). ACHN, HT29, MDA-MB-231, MCF7, and ZR75-1 xenograft mice were randomized into 4 groups (vehicle for everolimus (RAD001), vehicle for MLN0128, everolimus 10 mg/kg × 5 weekly (oral gavage), MLN0128 1 mg/kg × 5 weekly (oral gavage), n=8-10).Tumor volumes were calculated as previously described [54]. Mice were euthanized 24 hours after the last treatment and the tumors were flash-frozen.

### Statistical Analysis

For *in vitro* studies, comparisons between two groups were performed using the Student's t-test. All *in vitro* experiments were performed at least three times. For *in vivo* studies, comparisons between control and treatment groups were performed by using Mann-Whitney U. Data were presented as means ± SE.

## SUPPLEMENTARY MATERIAL, FIGURE AND TABLES


