# Supplementary material for: Catalytic mTOR inhibitors can overcome intrinsic and acquired resistance to allosteric mTOR inhibitors
Source: Oncotarget. 2014 Aug 10;5(18):8544–57. doi: 10.18632/oncotarget.2337 (PMC4226703; doi:10.18632/oncotarget.2337)
Supplement: Supplementary file 1 [file oncotarget-05-8544-s001.pdf]

Catalytic mTOR inhibitors can overcome intrinsic and acquired resistance to allosteric mTOR inhibitors

Supplementary Material

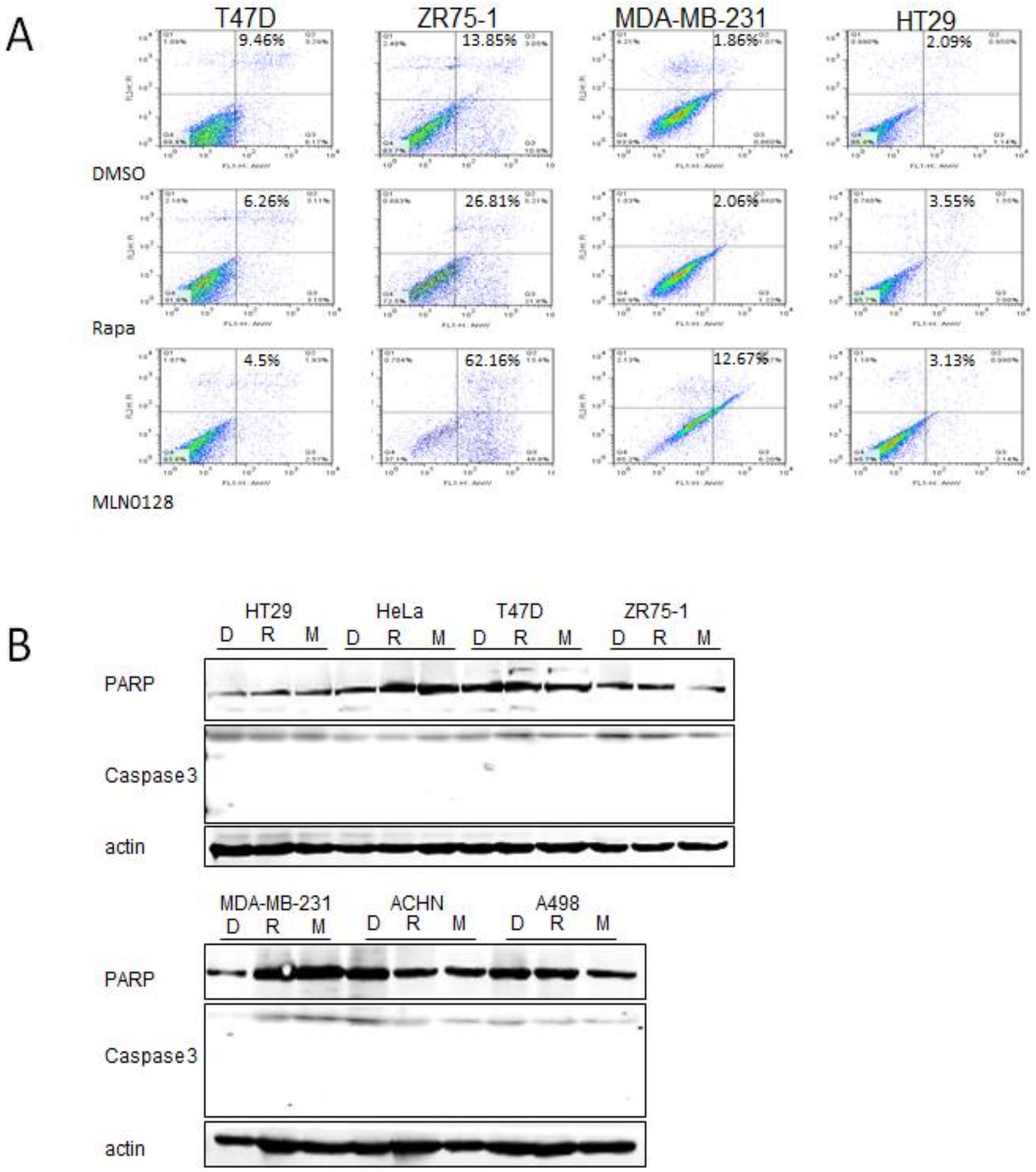

Supplementary Figure 1: (A) Percentages of annexin V positive cells. (B) Immunoblotting for apoptosis in cell lines.

Supplementary Table 1: Selected molecular characteristics of cell line panel.

Cell line	Tissue	ER	PR	HER2	Mutations
A498	Kidney				CDKN2A, SETD2, VHL
ACHN	Kidney				CDKN2A, NF2
BT474	Breast	Positive	Positive	Positive	PIK3CA, TP53
HCC70	Breast	Positive	Negative	Negative	PTEN, TP53
HCC1428	Breast	Positive	Positive	Negative	
HCC1806	Breast	Negative	Negative	Negative	CDKN2A, KDM6A, STK11, TP53
HT29	Colon				APC, BRAF, PIK3CA, SMAD4, TP53
M14	Skin				BRAF, CDKN2A, TP53
MCF7	Breast	Positive	Positive	Negative	CDKN2A, PIK3CA
MDA-MB-231	Breast	Negative	Negative	Negative	BRAF, CDKN2A, KRAS, NF2, TP53
MDA-MB-361	Breast	Positive	Positive	Positive	CDKN2A, PIK3CA
MDA-MB-435	Breast	Negative	Negative	Negative	FLT1, FLT4, IGF1R, PDK1
MDA-MB-468	Breast	Negative	Negative	Negative	PTEN, RB1, SMAD4, TP53
NCI/ADR-RES	Ovary				ERBB2, TP53
T47D	Breast	Positive	Positive	Negative	PIK3CA, TP53
ZR75-1	Breast	Positive	Negative	Negative	PTEN

References:

1. Stemke-Hale K, Gonzalez-Angulo AM, Lluch A, Neve RM, Kuo WL, Davies M, et al. An integrative genomic and proteomic analysis of PIK3CA, PTEN, and AKT mutations in breast cancer. Cancer Res. 2008;68:6084-91.

2. Forbes SA, Bindal N, Bamford S, Cole C, Kok CY, Beare D, et al. COSMIC: mining complete cancer genomes in the Catalogue of Somatic Mutations in Cancer. Nucleic Acids Res. 2011;39:D945-50.2.

Supplementary Table 2: List of genes assessed by targeted exome sequencing

Symbol	Chromosome region	Name
ABL1	chr9:133,710,831-133,763,060	c-abl oncogene 1, non-receptor tyrosine kinase
ACVR1B	chr12:52345486-52390859	activin A receptor, type IB
ADAMTS12	chr5:33,527,287-33,892,124	ADAM metallopeptidase with thrombospondin type 1 motif, 12
AKAP3	chr12:4,724,677-4,754,358	A kinase (PRKA) anchor protein 3
AKT1	chr14:105235689-105262080	v-akt murine thymoma viral oncogene homolog 1
ALK	chr2:29415641-30144432	anaplastic lymphoma kinase (Ki-1)
APC	chr5:112073556-112181935	adenomatous polyposis of the colon gene
AR	chrX:66763874-66944119	androgen receptor
ARAF	chrX:47420578-47431319	v-raf murine sarcoma 3611 viral oncogene homolog
ARID1A	chr1:27022522-27108601	AT rich interactive domain 1A (SWI-like)
ASXL1	chr20:30946153-31027121	additional sex combs like 1
ATM	chr11:108093559-108239826	ataxia telangiectasia mutated
ATR	chr3:142168078-142297668	ataxia telangiectasia and Rad3 related
ATRX	chrX:76760359-77041719	alpha thalassemia/mental retardation syndrome X-linked
AURKA	chr20:54944445-54967351	aurora kinas A
AURKB	chr17:8108050-8113883	aurora kinase B
BAI3	chr6:69345632-70099402	brain-specific angiogenesis inhibitor 3
BAP1	chr3:52435027-52444009	BRCA1 associated protein-1 (ubiquitin carboxy-terminal hydrolase)
BRAF	chr7:140433815-140624564	v-raf murine sarcoma viral oncogene homolog B1
BRCA1	chr17:41196313-41277500	familial breast/ovarian cancer gene 1
BRCA2	chr13:32889617-32973809	familial breast/ovarian cancer gene 2
CARD11	chr7:2945769-3083579	caspase recruitment domain family, member 11
CASP8	chr2:202122754-202152434	caspase 8, apoptosis-related cysteine peptidase
CBL	chr11:119076990-119178858	Cas-Br-M (murine) ecotropic retroviral transforming sequence
CD19	chr16:28943260-28950661	CD19 molecule
CDH1	chr16:68771195-68869444	cadherin 1, type 1, E-cadherin (epithelial) (ECAD)
CDH10	chr5:24487210-24644911	cadherin 10, type 2
CDH11	chr16:64980685-65155919	cyclin-dependent kinase 11
CDK4	chr12:58142005-58146164	cyclin-dependent kinase 4
CDK6	chr7:92234237-92465941	cyclin-dependent kinase 6
CDKN2A	chr9:21967752-21994490	cyclin-dependent kinase inhibitor 2A (p16(INK4a)) gene
CEBPA	chr19:33790842-33793430	CCAAT/enhancer binding protein (C/EBP), alpha
CHEK1	chr11:125496312-125525639	CHK1 checkpoint homolog (S. pombe)
CHEK2	chr22:29083731-29137822	CHK2 checkpoint homolog
COL14A1	chr8:121137352-121384266	collagen, type XIV, alpha 1
CPAMD8	chr19:17003763-17137625	C3 and PZP-like, alpha-2-macroglobulin domain containing 8
CREBBP	chr16:3775058-3930121	CREB binding protein (CBP)
CRIPAK	chr4:1385340-1389782	cysteine-rich PAK1 inhibitor
CSF1R	chr5:149432855-149492935	colony stimulating factor 1 receptor
CSMD1	chr8:2792876-4852328	CUB and Sushi muliple domain 1
CSMD2	chr1:33979609-34630875	CUB and Sushi multiple domains 2
CSMD3	chr8:113235161-114449242	CUB and Sushi muliple domain 3
CTNNB1	chr3:41240942-41281939	catenin (cadherin-associated protein), beta 1
CYLD	chr16:50775961-50835846	familial cylindromatosis gene
CYP2C19	chr10:96522463-96612670	cytochrome P450, family 2, subfamily C, polypeptide 19
DAXX	chr6:33,286,336-33,290,793	death-domain associated protein
DDR1	chr6:30856465-30867931	discoidin domain receptor tyrosine kinase 1
DDR2	chr1:162602228-162750237	discoidin domain receptor tyrosine kinase 2
DNMT3A	chr2:25455846-25564774	DNA (cytosine-5-)-methyltransferase 3 alpha
EGFR	chr7:55086725-55275030	epidermal growth factor receptor
ELN	chr7:73442427-73484234	elastin
EML4	chr2:42,396,490-42,559,686	echinoderm microtubule associated protein like 4
EP300	chr22:41488614-41576080	300 kd E1A-Binding protein gene
EPHA3	chr3:89156674-89531282	EPH receptor A3
ERBB2	chr17:37856254-37884914	v-erb-b2 erythroblastic leukemia viral oncogene homolog 2
ERBB3	chr12:56,473,892-56,497,127	v-erb-b2 erythoblastic lekemia viral oncogene homolog 3 (avian)
ERCC3	chr2:128014866-128051752	excision repair cross-complementing rodent repair deficiency, compl group 3
ERCC4	chr16:14014014-14046205	excision repair cross-complementing rodent repair deficiency, compl group 4
ERCC5	chr13:103459496-103524748	excision repair cross-complementing rodent repair deficiency, comp group 6
ESR1	chr6:152163859-152424408	estrogen Receptor 1
ETV5	chr3:185764108-185826901	ets variant 5
EZH2	chr7:148504475-148581414	cer of zeste homolog 2 (Drosophila)
FAM123B	chrX:63404998-63425624	family with sequence similarity 123B (FAM123B)
FAM135B	chr8:139142268-139509065	family with sequence similarity 135, member B
FAT3	chr11:92,085,262-92,629,633	fat tumor suppressor 3
FBXW7	chr4:153242411-153456185	F-box and WD-40 domain protein 7 (archipelago homolog, Drosophila)

FGFR1	chr8:38268657-38326352	FGFR1 oncogene partner (FOP)
FGFR2	chr10:123237845-123357972	fibroblast growth factor receptor 2
FGFR3	chr4:1795039-1810599	fibroblast growth factor receptor 3
FGFR4	chr5:176513921-176525124	fibroblast growth factor receptor 4
FLG	chr1:152274651-152297679	filaggrin
FLT1	chr13:28874483-29069265	fms-related tyrosine kinase 1 (VEGF/vascular permeability factor receptor)
FLT3	chr13:28577412-28674729	fms-related tyrosine kinase 3
FLT4	chr5:180028507-180076624	fms-related tyrosine kinase 4
FOXL2	chr3:138,663,067-138,665,982	forkhead box L2
GABRA6	chr5:161112658-161129598	GABA A receptor, alpha 6
GABRB3	chr15:26788695-27018251	GABA A1 receptor, beta 3
GATA1	chrX:48,644,982-48,652,715	GATA binding protein 3
GATA3	chr10:8096667-8117162	guanine monphosphate synthetase
GNA11	chr19:3094408-3121452	guanine nucleotide binding protein (G protein), alpha 11 (Gq class)
GNAQ	chr9:80335200-80646192	guanine nucleotide binding protein (G protein), q polypeptide
GNAS	chr20:57414795-57486249	guanine nucleotide binding protein (G protein), alpha stimulating activ polyp 1
HDAC9	chr7:18535885-19036984	histone deacetylase 9
HEATR7B2	chr5:40998123-41071444	HEAT repeat family member 7B2
HGF	chr7:81331445-81399452	hepatocyte growth factor
HMCN1	chr1:185703683-186160085	hemicentin 1
HNF1A	chr12:121416549-121440312	transcription factor 1, hepatic (HNF1)
HNF1B	chr17:36046435-36105096	HNF1 homeobox B
HRAS	chr11:532243-535550	v-Ha-ras Harvey rat sarcoma viral oncogene homolog
HYDIN	chr16:70841290-71264569	hydrocephalus inducing homolog
IDH1	chr2:209100954-209119806	isocitrate dehydrogenase 1 (NADP+), soluble
IDH2	chr15:90627214-90645708	isocitrate dehydrogenase 2 (NADP+), soluble
IGF1R	chr15:99192761-99507758	insulin-like growth factor 1 receptor
IKZF1	chr7:50344378-50472796	interleukin 21 receptor
IL6R	chr1:154377669-154440188	interleukin 6 receptor
IRS1	chr2:227596034-227663506	insulin receptor substrate 1
ITGA4	chr2:182321619-182402466	integrin alpha 4 (antigen CD49D subunit of VLA-4 receptor)
JAK1	chr1:65298906-65432187	Janus kinase 1
JAK2	chr9:4985245-5128182	Janus kinase 3
JAK3	chr19:17935595-17958841	Janus kinase 3
KCNB2	chr8:73449626-73850582	potassium voltage-gated channel, Shab-related subfamily, member 2
KDM6A	chrX:44732423-44971843	vascular endothelial growth factor receptor 2
KDR	chr4:55944427-55991762	vascular endothelial growth factor receptor 2
KIT	chr4:55524095-55606879	kallikrein-related peptidase 2
KRAS	chr12:25358180-25403854	v-Ki-ras2 Kirsten rat sarcoma 2 viral oncogene homolog
LAMA1	chr18:6941888-7117813	laminin, alpha 1
LPHN3	chr4:62362839-62938167	latrophilin 3
LRP1	chr12:57,522,282-57,607,123	low density lipoprotein receptor-related protein 1B
LRP1B	chr2:140988996-142889270	low density lipoprotein receptor-related protein 2
LRP2	chr2:169983620-170219122	low density lipoprotein receptor-related protein 3
MAP2K1	chr15:66679211-66783881	mitogen-activated protein kinase kinase 1
MAP2K4	chr17:11924135-12047050	mitogen-activated protein kinase kinase 4
MAP3K1	chr5:56110900-56191976	mitogen-activated protein kinase kinase kinase 1
MAP3K4	chr6:161412822-161538416	mitogen-activated protein kinase kinase kinase 4
MDN1	chr6:90353231-90529442	MDN1, midasin homolog
MECOM	chr3:168801287-169381563	MDS1 and EVI1 complex locus
MEN1	chr11:64570996-64578188	multiple endocrine neoplasia type 1 gene
MET	chr7:116312459-116438439	met proto-oncogene (hepatocyte growth factor receptor)
MITF	chr3:69788633-70017486	microphthalmia-associated transcription factor
MLH1	chr3:37034979-37092335	E.coli MutL homolog gene
MLL2	chr12:49412762-49449107	myeloid/lymphoid or mixed-lineage leukemia 2
MLL3	chr7:151832012-152133090	myeloid/lymphoid or mixed-lineage leukemia 3
MPL	chr1:43803475-43820134	myeloproliferative leukemia virus oncogene
MSH2	chr2:47630263-47710360	mutS homolog 2 (E. coli)
MSH6	chr2:48,010,221-48,034,084	mutS homolog 6 (E. coli)
MTOR	chr1:11166589-11322608	mammalian target of rapamycin complex 1
MYD88	chr3:38179969-38184510	myeloid differentiation primary response gene (88)
NAV3	chr12:78225069-78606788	neuron navigator 3
NCOR1	chr17:15935259-16118845	nuclear receptor corepressor 1
NF1	chr17:29,421,995-29,704,694	neurofibromatosis type 1 gene
NF2	chr22:29999545-30094583	neurofibromatosis type 2 gene
NFKB2	chr10:104154339-104162280	nuclear factor of kappa light polypeptide gene enhancer in B-cells 2
NOTCH1	chr9:139388897-139440238	Notch homolog 1, translocation-associated (Drosophila) (TAN1)
NOTCH2	chr1:120454178-120612276	Notch homolog 2
NOTCH3	chr19:15270445-15311792	notch 3
NOTCH4	chr6:32162621-32191844	notch4
NPM1	chr5:170814798-170837887	nucleophosmin (nucleolar phosphoprotein B23, numatrin)

NRAS	chr1:115247079-115259515	neuroblastoma RAS viral (v-ras) oncogene homolog
NSD1	chr5:176560833-176727213	nuclear receptor binding SET domain protein 1
PALB2	chr16:23614483-23652678	partner and localizer of BRCA2
PAPPA2	chr1:176432307-176811968	pappalysin 2
PAX5	chr9:36838531-37034476	paired box gene 5 (B-cell lineage specific activator protein)
PBRM1	chr3:52,579,368-52,713,739	polybromo 1
PCDH15	chr10:55580860-56561051	protocadherin 1
PCLO	chr7:82383321-82792197	piccolo (presynaptic cytomatrix protein)
PDGFRA	chr4:55095264-55164411	platelet-derived growth factor, alpha-receptor
PDGFRB	chr5:149493403-149535422	platelet-derived growth factor receptor, beta polypeptide
PIK3CA	chr3:178866311-178952495	phosphoinositide-3-kinase, catalytic, alpha polypeptide
PIK3CG	chr7:106505924-106547585	phosphoinositide-3-kinase, catalytic, gamma polypeptide
PIK3R1	chr5:67522118-67597647	phosphoinositide-3-kinase, regulatory subunit 1 (alpha)
PIKFYVE	chr2:209130991-209223474	protein phosphatase 2A activator, regulatory subunit 4
PKHD1	chr6:51480145-51952423	polycystic kidney and hepatic disease 1 (autosomal recessive)
PKHD1L1	chr8:110374706-110543499	polycystic kidney and hepatic disease 1 (autosomal recessive)-like 1
PPP1R3A	chr7:113516882-113559082	protein phosphatase 1, regulatory (inhibitor) subunit 3A
PPP2R1A	chr19:52693191-52729670	protein phosphatase 2, regulatory subunit A, alpha
PPP2R4	chr9:131873244-131911223	protein phosphatase 2A activator, regulatory subunit 4
PRDM1	chr6:106534195-106557814	PR domain containing 1, with ZNF domain
PRSS1	chr7:142457319-142460927	protease, serine, 1 (trypsin 1)
PTCH1	chr9:98205266-98270831	Homolog of Drosophila Patched gene1
PTEN	chr10:89623195-89728531	phosphatase and tensin homolog gene
PTK2	chr8:141668502-142011332	PTK2 protein tyrosine kinase 2
PTPN11	chr12:112856536-112947716	protein tyrosine phosphatase, non-receptor type 11
RAD51	chr15:40987327-41024354	RAD51 homolog
RAF1	chr3:12625102-12705700	v-raf-1 murine leukemia viral oncogene homolog 1
RB1	chr13:48877883-49056024	retinoblastoma gene
RELN	chr7:103112233-103629963	reelin
RET	chr10:43572517-43625795	ret proto-oncogene
RIMS2	chr8:104512976-105265451	regulating synaptic membrane exocytosis 2
RNF213	chr17:78313726-78370078	ring finger protein 213
RUNX1	chr21:36160099-36421595	runt-related transcription factor 1 (AML1)
RUNX1T1	chr8:92971152-93088365	runt-related transcription factor 1
RYR2	chr1:237205702-237997288	regulatory factor X, 2 (influences HLA class II expression)
SETD2	chr3:47057900-47205467	SET domain containing 2
SMAD4	chr18:48556583-48611409	SMAD family member 4
SMARCA4	chr19:11071598-11172959	SWI/SNF related, matrix assoc, actin dep reg of chrom, subfamily a, member 4
SMARCB1	chr22:24129150-24176704	SWI/SNF related, matrix assoc, actin dep reg of chrom, subfamily b, member 1
SMO	chr7:128828713-128853383	smoothened homolog (Drosophila)
SOS1	chr2:39208692-39347604	son of sevenless homolog 1
SPEN	chr1:16174359-16266950	spen homolog, transcriptional regulator
SPOP	chr17:47676248-47755525	speckle-type POZ protein
SPTA1	chr1:158580496-158656506	spectrin alpha, erythrocytic 1
STK11	chr19:1205798-1228434	serine/threonine kinase 11 gene (LKB1)
SYK	chr9:93564012-93660833	spleen tyrosine kinase
SYNE1	chr6:152442823-152958534	spectrin repeat containing, nuclear envelope 1
SYNE2	chr14:64319683-64693165	spectrin repeat containing, nuclear envelope 2
TBC1D4	chr13:75858809-76056250	TBC1 domain family, member 4
TET2	chr4:106067943-106200958	tet oncogene family member 2
TGFb1	chr19:41836651-41859816	transforming growth factor, beta 1
TGFBR2	chr3:30647994-30735631	transforming growth factor, beta receptor II
TNFAIP3	chr6:138188581-138204445	tumor necrosis factor, alpha-induced protein 3
TOP1	chr20:39657462-39753124	topoisomerase (DNA) I
TOP2A	chr17:38544798-38574169	topoisomerase II
TP53	chr17:7571720-7590863	tumor protein p53
TSC1	chr9:135766735-135820020	tuberous sclerosis 1
TSC2	chr16:2097990-2138712	tuberous sclerosis 2
TSHR	chr14:81421869-81612646	thyroid stimulating hormone receptor
USH2A	chr1:215796236-216596738	usher syndrome 2A
VHL	chr3:10183319-10193744	von Hippel-Lindau syndrome gene
WHSC1	chr4:1873123-1983933	Wolf-Hirschhorn syndrome candidate 1
WT1	chr11:32409325-32457087	Wilms tumour 1 gene
ZNF238	chr1:244214561-244220776	zinc finger protein 238
ZNF536	chr19:30863328-31048965	zinc finger protein 536
